# Water immersion tolerance by larval instars of stable fly, *Stomoxys calcitrans*, L1758 (Diptera: Muscidae) impairs the fitness performance of their subsequent stages

**DOI:** 10.1186/s12862-021-01810-z

**Published:** 2021-05-04

**Authors:** Steve B. S. Baleba

**Affiliations:** 1 International Centre of Insect Physiology and Ecology (icipe), P.O. Box 30772-00100, Nairobi, Kenya; 2 Present Address: Department of Evolutionary Neuroethology, Max Planck Institute for Chemical Ecology, Hans-Knöll-Straße 8, 07745 Jena, Germany

**Keywords:** *Stomoxys calcitrans*, Larval instars, Water immersion stress, Fitness parameters

## Abstract

**Background:**

In holometabolous insects, environmental factors experienced in pre-imaginal life stages affect the life-history traits within that stage and can also influence subsequent life stages. Here, I assessed tolerance to water immersion by the larval instars of the stable fly, *Stomoxys calcitrans* L. (Diptera: Muscidae) and its impact on the life-history traits of their subsequent life stages.

**Results:**

After submerging the three larval instars of *S. calcitrans* in distilled water, I found that the first instar larvae remained active for longer as compared to the second and third instar larvae. Also, the first instar larvae took a longer period to recover from the stress-induced immobility when removed from the water and returned to ambient temperature. When I followed the development of individuals of each larval instar that survived from water immersion, I found that their developmental time, weight, pupation percentage, adult emergence percentage and adult weight were negatively affected by this stressor. However, the weight of *S. calcitrans* adults developed from immersed first larval instar individuals was not affected by water immersion whereas their counterparts developed from immersed second and third larval instars had lower body weight. This suggests that in *S. calcitrans*, water immersion stress at the earlier stage is less detrimental than that experienced at late stages.

**Conclusion:**

This study provides a comparative overview of the fitness consequences associated with water immersion stress during *S. calcitrans* larval ontogeny. The results prove that the fitness shift induced by water immersion in *S. calcitrans* is stage-specific. My results illustrate the importance of considering each larval instar when assessing the impact of environmental factors on holometabolous insect performance as these may be decoupled by metamorphosis.

## Background

Insect metamorphosis is one of the well-known processes delimiting transitions between phenotypes. In holometaboly (complete metamorphosis), after the eggs hatch, larvae go through multiple moults (or ecdysis) in which the old cuticle is shed and a new one is produced enabling the insect to grow [1]. Insects undergoing this moulting process experience tremendous changes characterised by cell death which reshape or remove larval tissues, thus allowing adult body formation [2]. Larval moult is highly responsive to environmental conditions. Stress experienced during the preimaginal stage can influence metamorphosis, thereby carry-over and impact life-history parameters of the subsequent life stages [3]. These stressors include factors such as competition, temperature, and the nutritional value of developmental substrate. For instance, Baleba et al. [4] found that intra- and interspecific competition experienced by *Stomoxys calcitrans* (Diptera: Muscidae) larvae significantly reduces pupal weight, adult emergence and adult weight. In *Anopheles dalingi* (Diptera: Culicidae), larvae reared at 28 ℃ develop into adults with short wings as compared to their counterparts reared at 20 ℃ [5]. Morimoto et al. [6] found that in *Bactrocera tryoni* (Diptera: Tephritidae), larvae reared in a developmental substrate with lactose pupate and emerge less than those reared on substrates with sucrose and maltose. They also lead to adults with small body weight. In *Bactrocera minax* (Diptera: Tephritidae), an immersion of larvae in distilled water for more than 6 days later reduces their respiration, survival and pupation ratio [7].

Water is an important nutrient for the development and survival of insects. Maintaining its balance is a critical aspect of insect physiology [8]. The abnormal presence of water in the body of an insect can generate a long-lasting fitness consequence [9], specifically, in small-bodied insects possessing a high surface area to volume ratio [10]. In the order Diptera, the majority of studies assessing the impact of water stress on life-history traits have focused on the effects of its loss, namely desiccation (e.g. fruit flies [11–13], mosquitoes [14, 15], gall fly [16], drosophilids [17, 18]). These studies, however, fail to consider situations where, in sedentary and terrestrial pre-imaginal stages, the developmental substrate can be flooded during heavy rainfalls, leading to an excess of water inside the insect’s body (hyperhydration) and hypoxic or anoxic conditions. During hypoxia (or anoxia), insects are deprived of an adequate supply of oxygen which can cause severe consequences including inhibition of protein synthesis, lipid damages, increases of oxidative damage and decreases in ATP production [19]. This usually occurs in insects with immature stages developing in fallen rotten fruit (e.g. fruitflies), sand (e.g. tsetse flies), carrion (e.g. blowflies) or animal dung (e.g. house flies and stable flies). It is proposed that under such stressful conditions, these pre-imaginal stages enter a coma which is a state characterized by immobility, reduction in energy consumption and a lack of response to stimulation [20]. This suggests the existence of an adaptative strategy for surviving periodic and unpredictable water immersion. Although many insect species are probably well adapted to survive water immersion, the phenomenon is not well-documented and little is known of the behavioural and fitness consequence involved. In Diptera, a few studies conducted on phytophagous species have assessed the effect of water immersion on their survival. Duyck et al. [21] found that immersion duration (more than 6 h) significantly reduced the survival of *Ceratitis catoirii*, *Ceratitis rosa, Ceratitis capitata* and *Bactrocera zonata* (Diptera: Tephritidae). To my knowledge, no study has investigated the effect of pre-imaginal water immersion and its fitness consequence in hematophagous insects. Here, I studied the influence of this environmental stressor in *S. calcitrans* and its impact on their life history traits.

*Stomoxys calcitrans* is a blood-feeding fly distributed around the world that mechanically transmits viruses (e.g. West Nile Fever virus, Rift Valley Fever virus), bacteria (e.g. *Bacillus anthracis, Pasteurella multocida*), protozoans (e.g. *Trypanosoma evansi, Besnoitia besnoiti*), and helminths (e.g. *Habronema microstoma, Dirofilaria repens*) to their hosts. Hosts include cattle, camels, horses, dogs, and humans [22]. During their outbreak, *S. calcitrans* populations can induce weight loss in cattle by up to 19%, and lead to a 40% to 60% reduction in milk yields [23]. In the USA, Taylor et al. [24] estimated that *S. calcitrans* lead to economic losses of around $2.2 billion per year. Gravid females *S. calcitrans* lay their eggs on vertebrate herbivore dung [25] and rotting plant material such as silage, hay, grass clippings, and garden compost [26]. Larvae that emerge from these eggs mostly hatch after 24 h and go through three larval instars for 10 to 14 days before pupating. Being confined in the substrate where they have been deposited as eggs, this sedentary status permanently exposes *S. calcitrans* larvae to inundation during heavy rains. In such conditions, these larvae are likely to experience incomplete (hypoxia) or complete (anoxia) lack of oxygen. This is likely to disadvantage the development of larvae as female *S. calcitrans* avoid ovipositing in vertebrate dung with higher water content, which lead to poorer offspring performance [25].

The broad aim of this work was to study the tolerance to water immersion of the three larval instars of *S. calcitrans* and the fitness consequences evident in subsequent life stages. I predicted that the *S. calcitrans* larval instars that coped best with water immersion would then suffer more pronounced declines in fitness in later life stages. To test this, I assessed water immersion tolerance in the three larval instars of *S. calcitrans* by recording the time it took for them to enter into an immobile state after being immersed in water and the immobility recovery time. For the larvae that survived this immersion, I determined their life-history traits (Developmental time, larval weight, pupation rate, pupal weight, emergence percentage, emergence time and adult weight). This study provides information on how larval instars of *S. calcitrans* cope with water immersion and the fitness consequence that result from this stress. This knowledge provides an understanding of how *S. calcitrans* responds to environmental parameters.

## Methods

### Source of flies and culture maintenance

I obtained *S. calcitrans* larvae from the fourth generation of a previously established culture. To establish this culture, I collected wild individuals of *S. calcitrans* at the *icipe* Duduville campus in Nairobi, Kenya (1°13′12′' S, 36°52′48′' E; 1.600 m above sea level) using a Vavoua trap. I transferred trapped adults to cages (75 × 60 × 45 cm) in an insectary maintained at 25 ± 5 ℃ and 65 ± 5% relative humidity, with a 12L: 12D photocycle. I fed the flies two times per day (0800 and 1600 h) on defibrinated bovine blood on moistened cotton. When gravid, I presented the female flies with 1-week old fermented rabbit dung (placed in plastic containers: 21.5 × 14.5 × 7.4 cm) for oviposition. After exposure for 24 h, I transferred these containers to another cage (75 × 60 × 45 cm), and I monitored the development of the larval and pupal stages. I transferred the pupae to Petri dishes and introduced them to another cage for age-matched adult emergence. As described above, I provided blood and rearing media to the newly emerged adults. I continued this process until the fourth generation.

### Water immersion tolerance assay

**Fig. 1:**
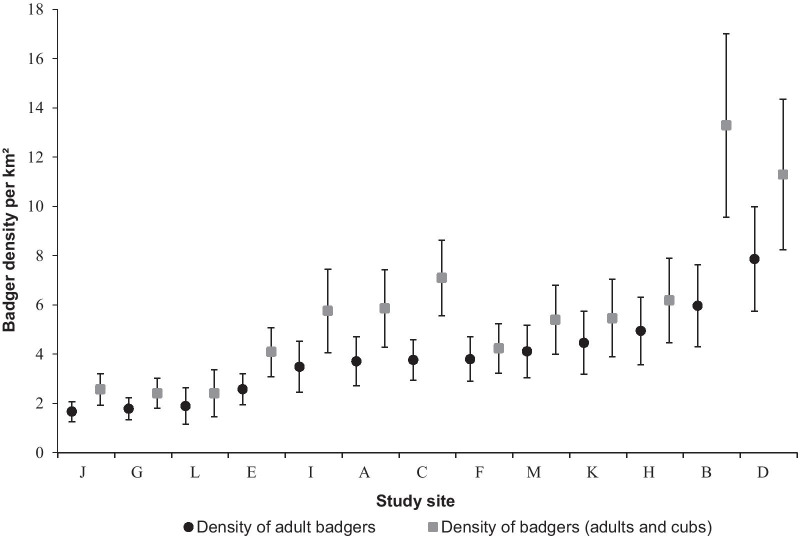
Overview of experimental methods. **a** Water immersion tolerance experiment: (1) L1, L2 and L3 larval instars of *S. calcitrans* reared on rabbit dung, (2) 30 L1, L2 and L3 larvae submerged individually in distilled water, (3) time recorded for L1, L2 and L3 individuals to enter into stress-induced immobility, (4) knocked-out larvae transferred to a paper towel, under ambient condition, (5) time recorded for L1, L2 and L3 individuals to recover from the immobility after being returned to the ambient temperature. **b** Fitness costs associated with water immersion: (1) L1, L2 and L3 larval instars of *S. calcitrans* reared on rabbit dung, (2) L1, L2 and L3 larvae transferred in distilled water to initiate immobility, (3) stressed larvae returned to rabbit dung, (4) life-history parameters recorded in stressed larvae after they recovered from the immobility. The clipart depicted in Fig. 1 is the product of my conception

I assessed tolerance to water immersion in the three larval instars of *S. calcitrans* by measuring the time it took to enter the immobile state after submersion in water and the time to recover from the immobility. To do so, with slight modifications, I followed the protocols of Benasayag-Meszaros [27] and Li et al. [7] as illustrated in Fig. 1a. I used larvae of 2, 4 and 6 days old, corresponding to the first, second, and third larval instars, respectively. I identified these developmental stages by inspecting the posterior spiracles, which are modified from round spiracular discs with two straight slits in the first instar to triangular discs with two and three sinuous slits in the second and third instars, respectively [28]. For each instar, I immersed 30 individuals singly in 35 ml of distilled water (25 ± 5 °C) contained in a Petri dish (size 55 mm × 15 mm) using soft forceps. I measured the time to enter into an immobile state time by recording the time from the immersion to cessation of larval movement. Attainment of immobility was noted when no movement was observed when the larva was gently touched with the forceps. The knocked-out larvae were removed from the water and placed on a paper towel (under ambient temperature) and the recovery time was measured as the time elapsed from this point to the first signs of larval body movement. I conducted these experiments under insectarium conditions of 25 ± 5 ℃ and 65 ± 5% relative humidity.

### Fitness cost after surviving from water immersion stress

I hypothesised that the *S. calcitrans* larval instars that recovered from the immobility induced by water immersion would suffer reduced performance in the life history traits of their subsequent life stages. To test this (see Fig. 1b), in a group of 10 individuals, I immersed the first, second and third larval instars of *S. calcitrans* separately in distilled water. For each group, I transferred immediately each individual that had entered into the immobile state into plastic cups filled with 50 g of 1-week old fermented rabbit dung and covered with a mesh sealed with a rubber band. I replicated this process 10 times on the same day. As a control treatment, I used the unimmersed larvae of each larval instar that sojourned out of the substrate (placed on a paper towel) as their counterpart immersed. To avoid introducing bias that could come from manipulation, I set up an individual and independent assay to record each of the following parameters: (1) larval developmental time (from the immersion day to pupation day), (2) larval weight, (3) pupation rate (equivalent to larval mortality), (4) pupal weight, (5) emergence percentage (equivalent to pupal mortality), (6) emergence time (from pupal to adult stage) and (7) adult weight. For the developmental parameter, I recorded the data on the first 50 individuals that reached the pupal stage (the first 5 individuals that pupated in each replicate). To record the larval weight data, per each replicate, I weighed 5 larvae randomly selected. I weighed individuals from the first larval instar at 4 and 10 days after immersion, individuals from the second larval stage at 2 and 5 days after immersion, and those from the third larval stage at 2 and 4 days after immersion. Also, for the pupae weight parameter, I weighed the first 5 pupae obtained in each replicate. Before weighing larvae and pupae, I gently cleaned each individual in distilled water to remove dung debris usually found on their body surface. Before weighing cleaned larvae, I allowed each individual to move on tissue paper for 15 to 20 s to remove water from their body. Also, before weighing cleaned pupae, I placed each individual on tissue paper to allow water absorption. I assessed the pupation and emergence percentage per replicate. To record the adult weight developed from each larval instar (50 individuals [first 5 individuals emerged from each replicate]), I used a cylindrical container (height: 4 cm; diameter: 3 cm) covered with a mesh to prevent their escape. After taring the container, I introduced a single adult to the tube and recorded its weight. Throughout the weighing process, I used a Sartorius balance (0.0001 g precision).

### Data analysis

I used the R environment for statistical computing (version 3.6.2) (R Core Team, 2019) for all statistical analyses and Adobe Illustrator CC 2017 (version 21.0) for graphical design. The time to enter into an immobile state data were normally distributed (Shapiro–Wilk test: *P* > *0.05*) and their variance between groups did not differ (Bartlett’s test: *P* < 0.05); therefore, using the R package called “userfriendlyscience” [29] to performed the Welch’s analysis of variance (ANOVA) test followed by the Games-Howell post hoc test to compare this time among the three *S. calcitrans* larval stages. Immobility recovery time data were not normally distributed (Shapiro–Wilk test: *P* < 0.05) and the variances were not homogeneous (Bartlett’s test: *P* < 0.05). Due to this, I used the Kruskal–Wallis test followed by Dunn’s post-hoc tests to determine whether the immobility recovery time differed among the *S. calcitrans* larval stages [30].

Developmental and emergence time data from the bioassay testing the effect of larval water immersion on the life history traits of subsequent stages were not normally distributed and their variances were heterogeneous. To compare these parameters between immersed and control larvae of each stage, I ran Mann–Whitney-Wilcoxon tests. Larval, pupal and adult weight data were normally distributed and their variances were homogeneous, therefore I used the unpaired t-test to see whether this parameter varied between individuals from immersed and unimmersed larvae. Owing to the binary nature of pupation (pupated vs. not pupated) and adult emergence (emerged vs. not emerged) data, I used generalized linear models (GLM) with binomial distribution to test whether these variables were affected by water immersion [31]. I established the significance of the model using analysis of deviance (with chi-squared test). All statistical comparisons were considered significant when *P* < 0.05.

## Results

### Water immersion tolerance

**Fig. 2:**
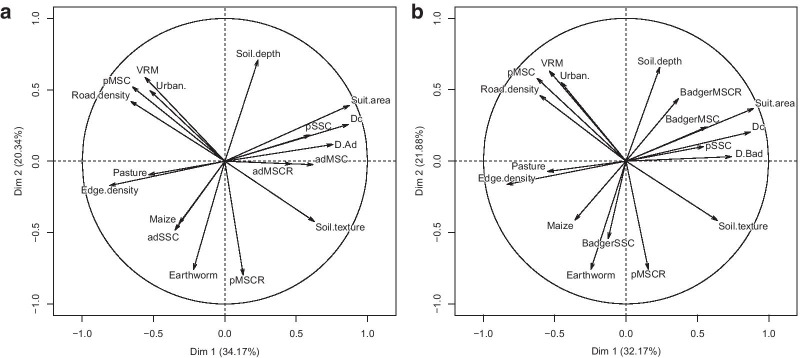
Water immersion tolerance in *S. calcitrans* larval instars. **a** Boxplot illustrating the variation in time taken to enter into an immobile state after water immersion (Welch’s ANOVA followed by Games-Howell post hoc test, *P* < *0.05*, n = 30). **b** Boxplot showing the variation in time taken to recover from the immobile state (Kruskal–Wallis test followed by Dunn’s post-hoc test, *P* < *0.05*, n = 30). Each box plots shows the median (bold horizontal lines) and whiskers the interquartile range. Yellow dots on each box plot represent individual data points. Treatments labelled with different lowercase letters are significantly different from each other

The time to enter into an immobile state significantly differed across the three *S. calcitrans* larval instars (Welch’s ANOVA: F_2, 55.95_ = 41.59, *P* < *0.0001*). After being immersed in water, first instar larvae took longer to lose their movements followed by second and third larval instars (Fig. 2a). Once returned to the air, the time taken to recover from the immobility induced by water immersion also significantly varied among the different larval instars (H = 35.80, df = 2, *P* < *0.0001*). Body movement resumed faster in the third and second larval instar in comparison with the first larval instar (Fig. 2b).

### Fitness cost of surviving water immersion

#### Individuals developing from first instar larvae

**Fig. 3:**
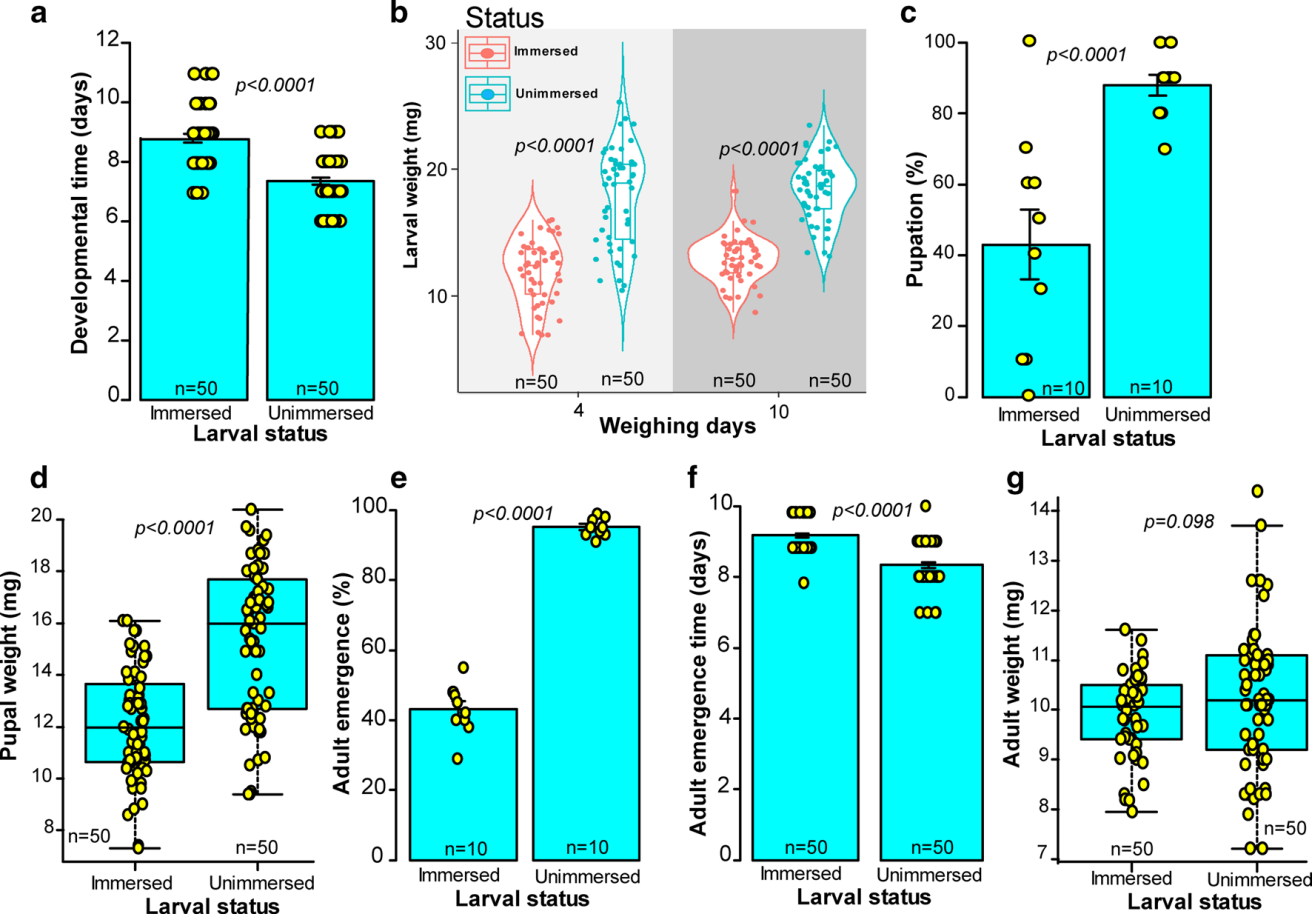
Life history parameters of *S. calcitrans* that were immersed (or unimmersed as a control) as first instar larvae. **a** Bar chart depicting the mean developmental time of immersed and unimmersed *S. calcitrans* (Wilcoxon–Mann–Whitney tests, *P* < *0.05*, n = 50). **b** Violin plots showing the weight of immersed and unimmersed *S. calcitrans* larvae at 4 and 10 days after water immersion (Unpaired t-test, *P* < *0.05*, n = 50). **c** Bar chart illustrating the mean percentage of immersed and unimmersed *S. calcitrans* that pupated (GLM with binomial distribution followed by the analysis of deviance test, *P* < *0.05*, n = 10). **d** Boxplot showing the difference in pupal weight between individuals that were immersed or unimmersed (Unpaired t-test, *P* < *0.05*, n = 50). **e** Bar chart depicting the mean percentage of immersed and unimmersed *S. calcitrans* that reached the adult stage (GLM with binomial distribution followed by the analysis of deviance test, *P* < *0.05*, n = 10). **f** Bar chart illustrating the mean emergence time in *S. calcitrans* individuals that were immersed and unimmersed (Wilcoxon–Mann–Whitney tests, *P* < *0.05*, n = 50). **g** Boxplot showing the variation of *S. calcitrans* adult weight from immersed and unimmersed individuals (Unpaired t-test, *P* < *0.05*, n = 50). On each bar chart, error bars indicate the standard error of the mean. Bar into each box shows the median and those at the extreme of the box shows the 25th–75th percentiles, which are extended by whiskers indicating 1.5 × the interquartile range from the 25th–75th percentiles. Coloured dots on each graph show data points from each replicate

Water immersion negatively affected larval developmental time (U = 2693, df = 1, *P* < *0.0001*), larval weight (Day4: t = − 9.07, df = 85.5, *P* < *0.0001*; Day 10: t = − 12.72, df = 88.73 *P* < *0.0001*), pupation (GLM: χ^2^ = 19.89, df = 1, *P* < *0.0001*), pupal weight (t = − 7.17, df = 116.45, *P* < *0.0001*), adult emergence (GLM: χ^2^ = 401.41, df = 1, *P* < *0.0001*), and pupal development time (U = 2517, df = 1, *P* < *0.0001*) in the first instar larvae of *S. calcitrans* that survived this environmental stressor. Compared with unimmersed larvae, immersed larvae developed slowly (Fig. 3a) and had low larval weight (Fig. 3b), pupation (Fig. 3c), pupal weight (Fig. 3d) and adult emergence (Fig. 3e). Also, these larvae took longer to emerge from their puparia (Fig. 3f). Only adult weight was not significantly affected by water immersion (Fig. 3g, t = − 1.67, df = 109.11, *P* = *0.098*).

#### Individuals developing from second instar larvae

**Fig. 4:**
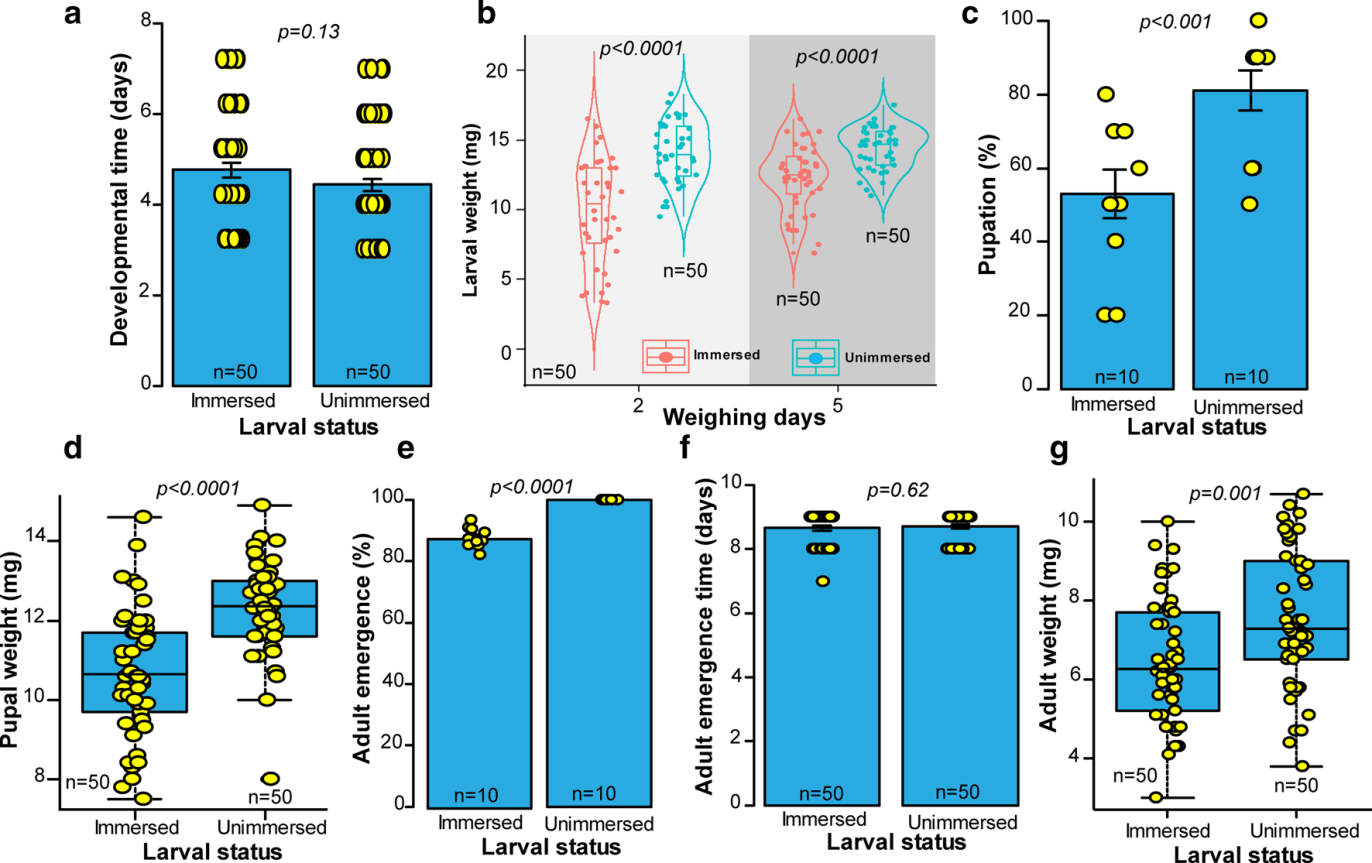
Life history parameters of *S. calcitrans* that were immersed (or unimmersed as a control) as second instar larvae. **a** Bar chart depicting the mean developmental time of immersed and unimmersed *S. calcitrans* (Wilcoxon–Mann–Whitney tests, *P* < *0.05*, n = 50). **b** Violin plots showing the weight of immersed and unimmersed *S. calcitrans* larvae at 4 and 10 days after water immersion (Unpaired t-test, *P* < *0.05*, n = 50). **c** Bar chart illustrating the mean percentage of immersed and unimmersed *S. calcitrans* that pupated (GLM with binomial distribution followed by the analysis of deviance test, *P* < *0.05*, n = 10). **d** Boxplot showing the difference in pupal weight between individuals that were immersed or unimmersed (Unpaired t-test, *P* < *0.05*, n = 50). **e** Bar chart depicting the mean percentage of immersed and unimmersed *S. calcitrans* that reached the adult stage (GLM with binomial distribution followed by the analysis of deviance test, *P* < *0.05*, n = 10). **f** Bar chart illustrating the mean emergence time in *S. calcitrans* individuals that were immersed and unimmersed (Wilcoxon–Mann–Whitney tests, *P* < *0.05*, n = 50). **g** Boxplot showing the variation of *S. calcitrans* adult weight from immersed and unimmersed individuals (Unpaired t-test, *P* < *0.05*, n = 50). On each bar chart, error bars indicate the standard error of the mean. Bar into each box shows the median and those at the extreme of the box shows the 25th–75th percentiles, which are extended by whiskers indicating 1.5 × the interquartile range from the 25th–75th percentiles. Coloured dots on each graph show data points from each replicate

With the exception of larval development time (Fig. 4a; U = 2771.5, *P* = *0.131*) and pupal development time (U = 1193, *P* = *0.62*), other fitness parameters including the larval weight (Fig. 4b: Day 2: t = − 6.01, df = 63.39, *P* < *0.0001*, Day 3: t = − 4.93, df = 74.03, *P* < *0.0001*), pupation (Fig. 4c: GLM: χ^2^ = 7.35, df = 1, *P* < *0.05*), pupal weight (Fig. 4d: t = − 5.64, df = 91.01, *P* < *0.0001*), adult emergence (Fig. 4e: t = − 15.64, df = 66.21, *P* < *0.0001*) and adult weight (Fig. 4g: t = − 3.3593, df = 97.003, *P* = *0.001*) were significantly greater in unimmersed larvae compared with those immersed in water.

#### Individuals developing from third instar larvae

**Fig. 5:**
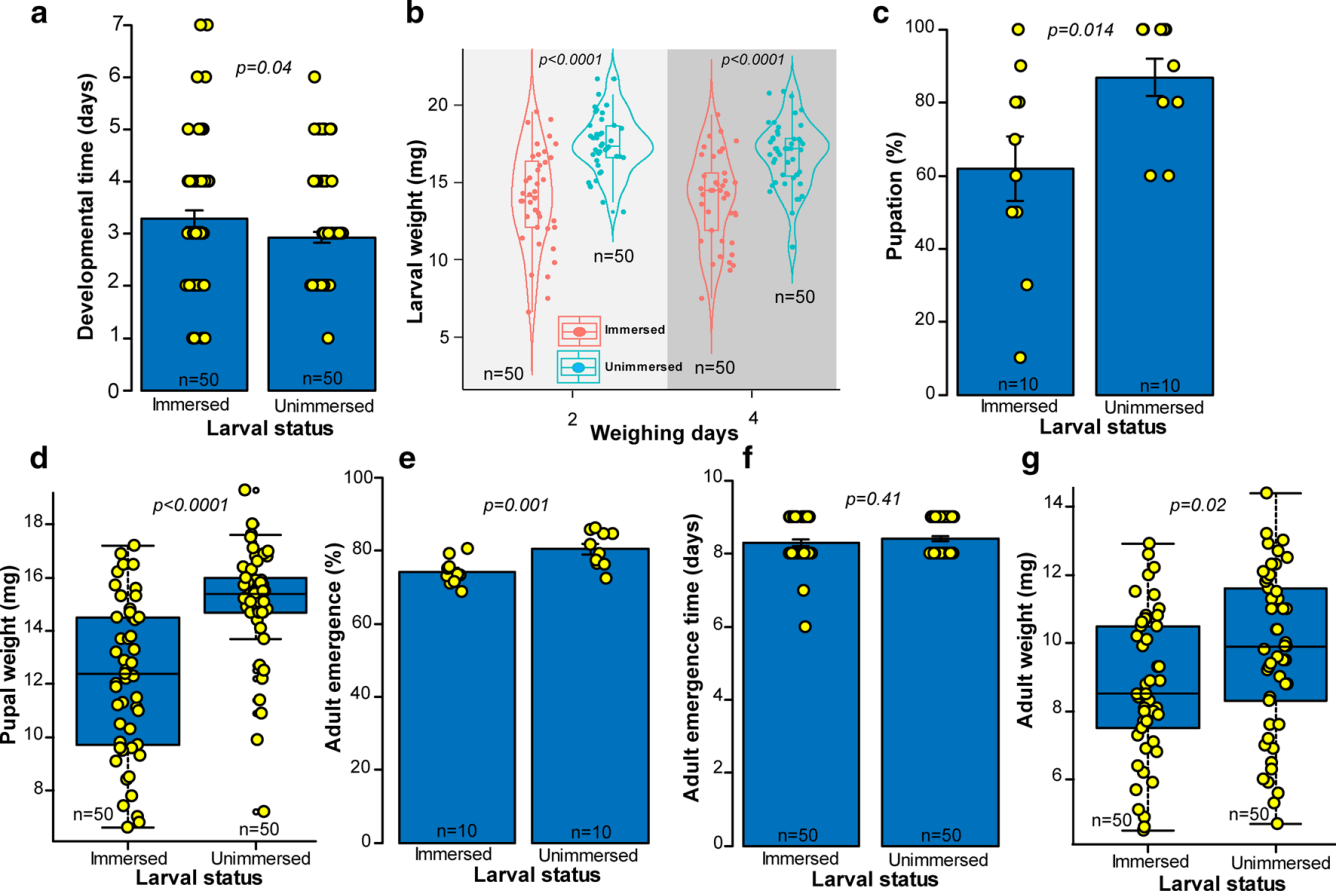
Life history parameters of *S. calcitrans* that were immersed (or unimmersed as a control) as third instar larvae. **a** Bar chart depicting the mean developmental time of immersed and unimmersed *S. calcitrans* (Wilcoxon–Mann–Whitney tests, *P* < *0.05*, n = 50). **b** Violin plots showing the weight of immersed and unimmersed *S. calcitrans* larvae at 2 and 5 days after water immersion (Unpaired t-test, *P* < *0.05*, n = 50). **c** Bar chart illustrating the mean percentage of immersed and unimmersed *S. calcitrans* that pupated (GLM with binomial distribution followed by the analysis of deviance test, *P* < *0.05*, n = 10). **d** Boxplot showing the difference in pupal weight between individuals that were immersed or unimmersed (Unpaired t-test, *P* < *0.05*, n = 50). **e** Bar chart depicting the mean percentage of immersed and unimmersed *S. calcitrans* that reached the adult stage (GLM with binomial distribution followed by the analysis of deviance test, *P* < *0.05*, n = 10). **f** Bar chart illustrating the mean emergence time in *S. calcitrans* individuals that were immersed and unimmersed (Wilcoxon–Mann–Whitney tests, *P* < *0.05*, n = 50). **g** Boxplot showing the variation of *S. calcitrans* adult weight from immersed and unimmersed individuals (Unpaired t-test, *P* < *0.05*, n = 50). On each bar chart, error bars indicate the standard error of the mean (SEM). Bar into each box shows the median and those at the extremes of the box shows the 25th–75th percentiles, which are extended by whiskers indicating 1.5 × the interquartile range from the 25th–75th percentiles. Coloured dots on each graph illustrates individual data points

Water immersion significantly affected the life history traits of the subsequent stages of *S. calcitrans* larvae immersed during the third instar. Individuals developing from immersed third instar larvae took longer to complete larval development (Fig. 5a: U = 3653.5, df = 1, *P* = *0.04*) and their larval weight was suppressed (Fig. 4b: Day 2: t = − 6.06, df = 64.51, *P* < *0.0001*; Day 4: t = − 4.99, df = 75.53, *P* < *0.0001*) in comparison with those that were unimmersed. In addition, fewer pupated (Fig. 5c: GLM: χ^2^ = 6.03, df = 1, *P* = *0.014*), and their pupal weight (Fig. 5d: t = − 5.69, df = 88.69, *P* < *0.0001*), adult emergence (Fig. 5e: GLM: χ^2^ = 10.23, df = 1, *P* = *0.001*) and adult weight (Fig. 5g: t = − 2.32, df = 96.72, *P* = *0.02*) were lower than unimmersed counterparts. Only the pupal development time was similar between the two treatments (Fig. 5f: U = 1146, df = 1, *P* = *0.40*).

## Discussion

In the present study, I aimed to study water immersion resistance in *S. calcitrans* larvae and the fitness consequences of immersion. I found that the three larval instars of *S. calcitrans* had different levels of tolerance to water immersion. After water immersion, the first larval instar of *S. calcitrans* took the longest to enter stress-related immobility followed by the second and third larval instars. This suggests that the first larval instar of *S. calcitrans* is less sensitive to water immersion stress than later instars. Callier et al. [32] stated that when trapped in water, insects are likely to encounter hypoxic or anoxic conditions. Following this, first instar larvae of *S. calcitrans* are more tolerant of the lack of oxygen (anoxic or hypoxic conditions). Also, Callier et al. [33] explained that as an insect grows, its increase in mass is associated with an elevated metabolic rate and hence oxygen demand. The results are consistent with those of Callier & Nijhout [34] and Heinrich et al. [35] who, respectively, found that in *Manduca sexta* (Lepidoptera: Sphingidae) and *Drosophila melanogaster* (Diptera: Drosophilidae), the late larval instars are more susceptible to a lack of oxygen than earlier ones. I also found that the immobility recovery time of first instar larvae was longer than for other instars. This may be because the recovery time is positively correlated with the time needed to induce immobility [36, 37] owing to the relative metabolic disturbance during stress [38].

After immersing the three larval instars of *S. calcitrans* in water, most of the life-history traits of the individuals that developed from these larvae were negatively impacted. These individuals experienced slow growth, and weighed, pupated and emerged less, in comparison with those from unimmersed larvae. The lack of atmospheric oxygen can constitute a powerful stressor on insect development and survival [39]. I propose that this factor could explain the reduction of the fitness parameters in the subsequent stages of immersed *S. calcitrans* larval instars. Hypoxia generally lengthens development, decreases body size and growth rate, and survival of insects (reviewed by Harrison et al. [40, 41]). This has been demonstrated in the beetle *Tenebrio molitor* (Coleoptera: Tenebrioridae) [42], *M. sexta* [43] and *D. melanogaster* [44]. My study does not corroborate the traditional conjecture that metamorphosis is an adaptative process separating traits between life stages, allowing evolutionary independence of pre-and post-metamorphic phenotypes [45]. Indeed, water immersion stress in the three larval instars of *S. calcitrans* significantly affected the fitness parameters of the following life stages.

I found that water immersion stress of first instar larvae had detrimental effects on adult body size of lower magnitude than when occurring during the second or third instar. Only adults developing from immersed first instar larvae had the same weight as those developing from their unimmersed counterparts. This suggests that adults developing from immersed first instar larvae might have enough time to repair and compensate for the stress induced by water immersion. It may be that the stage-specific stress effects on the body of insect adult depend on whether enough time has elapsed for recovery to occur [46]. This result implies that stress at an earlier stage is less detrimental than that applied at late stages, and this finding is consistent with those from other insects. For instance, Heinrich et al. [35] found that in *D. melanogaster,* exposure to hypoxia at the late stage (third larval instar and early pupal stage) significantly reduced adult mass. In *Plutella xylostella* (Lepidoptera: Plutellidae), heat stress at earlier developmental stages was less detrimental for adult reproduction than heat stress experienced at later developmental stages [46]. The body size and coloration of *Harmonia axyridis* (Coleoptera: Coccinellidae) is affected by heat stress at the fourth-instar larval or pupal stages but not at early development stages [47]. In the butterfly *Bicyclus anynana* (Lepidoptera: Satyridae), the wing pattern is sensitive only to the late larval stage temperatures [48]. This increase of sensitivity during insect growth is ecologically adaptive since the late larval environment is the most accurate predictor for the adult environment.

In summary, my study reveals that immersion of *S. calcitrans* larvae in water impairs the life history traits of their subsequent life stages. I demonstrated that the developmental periods during which this stress occurs in *S. calcitrans* can affect the fitness parameters of later life stages. Exposure to water immersion stress during the first larval instar did not impact the weight of adults developing from these larvae, whereas this effect was observed in adults developing from the immersed second and third larval instars. My results highlight the importance of considering ontogeny when studying the impact of environmental stressors on the fitness of holometabolous insects.

## Data Availability

The datasets used and/or analysed during the current study available from the corresponding author on reasonable request.

## Abbreviations

ANOVA: Analysis of variance
L: Light
D: Dark
m: Meter
cm: Centimetre
^°^C: Degree Celsius
g: Gram
